# Surveillance for Hepatocellular Carcinoma in Patients with NASH

**DOI:** 10.3390/diagnostics6020022

**Published:** 2016-06-07

**Authors:** Philippe Kolly, Jean-François Dufour

**Affiliations:** 1 Hepatology, Department of Clinical Research, University of Bern, 3010 Bern, Switzerland; philippe.kolly@insel.ch; 2 University Clinic for Visceral Surgery and Medicine, Inselspital, University of Bern, 3010 Bern, Switzerland

**Keywords:** HCC, NASH, NAFLD, surveillance, screening

## Abstract

European and American guidelines recommend surveillance for hepatocellular carcinoma (HCC) by performing ultrasonography on a six-month basis on an at risk population, defined by presence of cirrhosis. HCC, due to non-alcoholic steatohepatitis (NASH), is rising. Patients with NASH have a high risk of developing HCC and, therefore, have to be enrolled in a screening program. One of the challenges with NASH-induced HCC is that half of the cases arise in non-cirrhotic patients. There is a need to identify those patients in order to screen them for HCC. The obesity of these patients is another challenge, it makes ultrasound screening more difficult. Other radiological methods, such as computer tomography (CT) scans or magnetic resonance imaging (MRI), are available, but the surveillance program would no longer be cost-effective. There is a need to prospectively acquire information on cohorts of patients with NASH in order to improve the tools we have to diagnose early tumors in these patients.

## 1. Introduction

In oncology, surveillance means enrollment in a program where a screening test is regularly applied to an at risk population. The goal is to identify cancers at an early stage, which can be better treated and—other as consequence—improves the outcome of the patients. For hepatocellular carcinoma (HCC), American and European guidelines have a clear position regarding surveillance [1,2]. The screening test is ultrasonography of the liver, applied on a six-month basis to a population at risk, defined by the presence of cirrhosis. In areas where the hepatitis B virus (HBV) is endemic and where the carcinogen aflatoxin can be found, the recommendations are to screen HBV positive patients, starting at defined ages, independent of the presence of cirrhosis. Two randomized controlled trials (RCT) performed in Asia aimed to assess the outcomes of a surveillance program. The first compared screening, by measuring alphafetoprotein (AFP) and performing ultrasonography against no screening in HBV-infected Chinese patients and found a reduction of mortality by 37% in the screened group, mostly because more patients could undergo surgical resection [3]. However, this study has some limitations, as adherence to the screening program was suboptimal (58%) and the study does not give any information about the level of fibrosis in the liver. The second study was performed in HBV-infected men with a six-month interval AFP screening. Although they found a significantly higher proportion of early HCC in the surveillance group (29.6% against 6.0%), there was no difference in the five-year survival between groups [4]. This study also had a low compliance (below 50%) and only used AFP. The use of AFP as a screening test is controversial, since many HCCs will never produce AFP and it can be elevated in patients with cirrhosis without tumor. These two studies are far from perfect, but the field has evolved so much that there will very likely be no better RCT to support regular screening in a population at risk for HCC. Nonetheless, a recent meta-analysis, based on 47 retrospective and prospective studies, showed that HCC surveillance is associated with improved early tumor detection, receipt of curative therapy, and overall survival in patients with cirrhosis [5].

## 2. The Incidence of NASH-Induced HCC Is Growing

The number of patients affected by HCC is increasing [6], probably for several reasons: Better surveillance of patients at risk, the aging cohort of hepatitis C (HCV)-infected patients, and the increasing importance of non-alcoholic steatohepatitis (NASH) in the landscape of liver diseases [7]. NASH can progress to cirrhosis and to HCC [8,9,10] and, therefore, patients with cirrhosis due to NASH have to be enrolled in a surveillance program for HCC. One has to recognize that histological diagnosis of a NASH is difficult at the stage of cirrhosis, since characteristic features disappear at this stage. The majority of cryptogenic cirrhosis is actually burned out NASH [11,12].

Patients with NASH are at particular risk to develop HCC. They have clinical abnormalities linked to this type of tumor. Obesity in young adults (mid-20s to mid-40s) has been identified as a risk factor (OR = 2.6) for HCC in a case-control study in the United States [13]. Even obesity in childhood (between 7 and 13 years old) has been reported to be associated with HCC later in life in a retrospective cohort of 285,884 Danish boys and girls [14]. Obesity increases the risk for many cancers, but the odd ratio is particularly high for HCC. In a prospective study comparing relative risk for various cancers in men with normal weight against men with a BMI of >35, liver cancer had the highest relative risk (RR = 4.25) [15]. In a cohort of 30,201 patients who underwent liver transplantation from the United Network of Organ Sharing (UNOS), obesity was independently predictive for HCC in cryptogenic cirrhosis (OR = 11.1) [16]. Diabetes mellitus has been, in many studies, associated with HCC in retrospective, as well as in prospective, studies. In the United States, a study showed an increased risk for HCC (RR = 2.13) by following 216,831 patients diagnosed with diabetes [17]. An analysis of a retrospective cohort with 153,852 Swedish patients with diabetes showed a four-fold increased risk for primary liver cancer during a 1–24 year follow-up [18]. A prospective study in Asia showed that obesity and diabetes had synergistic effects in hepatitis-infected patients, with a 100-fold increased risk for HCC [19]. Interestingly, due to the high prevalence of diabetes and obesity in the United States, even if the OR for HCC is far less for diabetes and/or obesity (OR = 2.47) than for other risk factors, such as HCV (OR = 39.89), those factors have the greatest population attributable fraction. This means that the elimination of diabetes and/or obesity could reduce the incidence of HCC, more than the elimination of any other factor [20]. With that objective in mind, studies showed that the risk factors for HCC, represented by diabetes mellitus, could be mitigated. Firstly, antidiabetic drugs like metformin could lower the risk of developing HCC. A meta-analysis based on three cohort studies and four case-control studies showed a significantly-reduced risk of HCC in diabetic patients using metformin [21]. Secondly, lifestyle modifications could also prevent the development of HCC, as has been shown in animals [22].

## 3. Identifying and Screening Patients with NASH

It appears that in the last few years, HCC, in the context of NASH, arises in half of the cases before cirrhosis. In a pathological analysis of 31 resected HCC patients in France with metabolic syndrome as the only risk factor, most of the patients had a no significant fibrosis [23]. In Japan, pathological analysis of 87 NASH patients with HCC revealed that only 51% of the patients had liver cirrhosis, with a lower prevalence among men compared with women (39% *versus* 70%) [24]. In a retrospective analysis of 120 HCC patients with non-alcoholic fatty liver disease (NAFLD) in the United States, 58.3% of them had cirrhosis [25]. Patients with NASH-induced HCC often present with large tumors and have a worse prognosis than those with other etiologies. In a retrospective analysis of the Surveillance, Epidemiology and End Results (SEER)-Medicare database, 50% of viral-hepatitis-infected patients with HCC died after one year, whereas 61% of the NAFLD patients with HCC died after one year [26]. This can be due to the fact that they are older, that they often have comorbidities [27], and also that these patients have not been involved or enrolled in a surveillance program. Indeed, since about half of patients with NASH develop HCC before cirrhosis, these patients will not be enrolled in a surveillance program. A retrospective analysis of a cohort with 1500 HCC patients from Veterans Administration (VA) hospitals showed that patients with NAFLD-HCC underwent less-frequent surveillance (43.3%) in the three years before HCC diagnosis than patients with alcohol- (59.8%) or HCV-induced (86.7%) HCC [25]. This raises a very important challenge for research in the next years. We need to identify patients with NASH at risk of developing HCC, and those who are not, to respectively enroll them in a surveillance program or not. We need to discover these parameters, which can be circulating biomarkers, which can be genetic markers, or a combination of both. The variation in the PNPLA3 gene has been associated with steatohepatitis, with fibrosis, cirrhosis, and also HCC. In a study comparing genotype frequencies between NAFLD-HCC cases and NAFLD-controls, the carriage of the PNPLA3 rs738409 C > G polymorphism increased the risk for HCC [28]. This single nucleotide polymorphism (SNP) can be one tool in helping to stratify patients. This tool is not perfect, and it needs to be supplemented with other parameters.

Another challenge of surveillance in patients with NASH is due to the fact that obesity compromises the completeness of an ultrasound examination of the liver. In these patients, it is difficult to examine all the segments of the liver. An Italian study showed that a BMI > 25 was significantly associated with ultrasound surveillance failure [29]. For this reason, it is not rare to have to perform CT scans or MRIs to get better images of the liver in these patients. This complicates surveillance and also increases costs. It has been shown that surveillance programs by application of regular sonographies in patients at risk for HCC are cost effective. However, this would no longer be the case if MRIs or CT scans have to be performed [30]. A study based on cirrhotic patients suggested that AFP has a better sensitivity and specificity in NAFLD patients than in HCV-patients, with a sensitivity of 89.7% and a specificity of 85.1% at a cut-off value of 20 ng/mL in NAFLD patients [31]. According to the low number of NAFLD patients with HCC in that study (*n* = 39), this result has to be assessed in a bigger sample, which also contains non-cirrhotic patients. Nonetheless, also here, identification of biomarkers with a better accuracy than alpha-fetoprotein is urgently needed.

## 4. Conclusions

The challenge for physicians engaged in the treatment of patients with NASH will be in the coming years, to earlier diagnose HCC in patients at particular risk. We need to improve the tools that we have to do that. In order to achieve this goal, we need to prospectively acquire information on cohorts of patients with NASH. This is the only way to propose an earlier diagnosis for better treatment and longer survival in these patients.
